# The latest edition of WHO and ELN guidance and a new risk model for Chinese acute myeloid leukemia patients

**DOI:** 10.3389/fmed.2023.1165445

**Published:** 2023-06-23

**Authors:** Xiaoning Wang, Jie Wang, Suhua Wei, Juan Zhao, Beibei Xin, Guoqing Li, Jing Zhao, Di Wu, Minna Luo, Sijie Zhao, Ying Chen, Haibo Liu, Hailing Zhang, Jingcheng Wang, Wenjuan Wang, Huaiyu Wang, Hui Xiong, Pengcheng He

**Affiliations:** ^1^Department of Hematology, The First Affiliated Hospital of Xi'an Jiaotong University, Xi'an, China; ^2^Shanghai Yuanqi Biomedical Technology Co., Ltd., Shanghai, China; ^3^National Clinical Research Center for Hematologic Diseases, The First Affiliated Hospital of Soochow University, Suzhou, China

**Keywords:** acute myeloid leukemia, World Health Organization, European Leukemia Net, risk model, prognosis

## Abstract

**Objective:**

Diagnosis classification and risk stratification are crucial in the prognosis prediction and treatment selection of acute myeloid leukemia (AML). Here, we used a database of 536 AML patients to compare the 4th and 5th WHO classifications and the 2017 and 2022 versions of ELN guidance.

**Methods:**

AML patients were classified according to the 4th and 5th WHO classifications, as well as the 2017 and 2022 versions of the European LeukemiaNet (ELN) guidance. Kaplan–Meier curves with log-rank tests were used for survival analysis.

**Results:**

The biggest change was that 25 (5.2%), 8 (1.6%), and 1 (0.2%) patients in the AML, not otherwise specified (NOS) group according to the 4th WHO classification, were re-classified into the AML-MR (myelodysplasia-related), KMT2A rearrangement, and NUP98 rearrangement subgroups based on the 5th WHO classification. Referring to the ELN guidance, 16 patients in the favorable group, six patients in the adverse group, and 13 patients in the intermediate group based on the 2017 ELN guidance were re-classified to the intermediate and adverse groups based on the 2022 ELN guidance. Regrettably, the Kaplan–Meier curves showed that the survival of intermediate and adverse groups could not be distinguished well according to either the 2017 or 2022 ELN guidance. To this end, we constructed a risk model for Chinese AML patients, in which the clinical information (age and gender), gene mutations (*NPM1, RUNX1, SH2B3*, and *TP53*), and fusions (CBFB::MYH11 and RUNX1::RUNX1T1) were included, and our model could help divide the patients into favorable, intermediate, and adverse groups.

**Conclusion:**

These results affirmed the clinical value of both WHO and ELN, but a more suitable prognosis model should be established in Chinese cohorts, such as the models we proposed.

## Introduction

Acute myeloid leukemia (AML) represents the most common type of acute leukemia in adults worldwide (1). Accumulated evidence has revealed that genetic abnormalities, such as gene mutations and fusions play crucial roles in the pathogenesis of AML, causing hyperproliferation and maturational arrest of myeloid precursor cells (2, 3). The 4th revision of the World Health Organization (WHO) classification of hematologic malignancies divides AML into 11 subgroups based on genetic abnormalities (4). Recently, the 5th revision of the WHO classification was published (5), and several alterations were made based on AML, such as the subgroups of *KMT2A*/*MECOM*/*NUP98* rearrangement and *CEBPA* mutation, as well as the AML, myelodysplasia-related (MR) subgroup.

In the present study, we used a dataset of 536 consecutive subjects with AML initially diagnosed using the 2016 WHO criteria to compare how these subjects would be classified using the 2022 WHO criteria. In addition, we compared the prognostic classification of AML according to the 2017 and 2022 versions of the European Leukemia Net (ELN) guidelines (6, 7) and established a new prognostic model including clinical information, gene mutations, and fusions for Chinese AML.

## Patients and methods

### Patients

A total of 536 patients with primary AML were included in this study between September 2013 and February 2021 from the First Affiliated Hospital of Xi'an Jiaotong University. The patients were diagnosed with AML according to the 4th or 5th version of the WHO guidance (4, 5) and followed up until July 2022. General clinical characteristics [age, sex, the proportion of blasts in bone marrow (BM) samples, karyotype, white blood cell (WBC) counts, red blood cell (RBC) counts, hemoglobin, blood platelet counts (PLT), activated partial thromboplastin time (APTT), prothrombin time, thrombin time, fibrinogen, fibrinogen degradation product, D-dimer, and survival time], structural variations [5/5q deletion (-5/-5q),−7/-7q, inv (involvement) (16)(p13q22), t (translocation) (16;16)(p13;q22), t(8;21)(q22;q22), t(9;22)(q34.1;q11.2), inv(3)(p21p26), and t(3;3)(q21;q26)], and fusion genes (*CBFB-MYH11, BCR-ABL1, KMT2A-PARTNER, TLS-ERG, PML-RARA, RUNX1-RUNXT1, NUP98-PARTNER, DEK-NUP214, FIPIL1-PDGFR*α, *AML1-ETO, NPM1-RAR*α, *PLZF-RAR*α, and *SET-NUP214*) were collected and shown as Table 1. After manual evaluation of the karyotype, fusion, and mutation, patients were assigned to respective 4th/5th WHO and ELN 2017/2022 risk groups. This study was approved by the First Affiliated Hospital of Xi'an Jiaotong University. Informed consent forms were signed by each patient.

**Table 1:** Baseline characteristics.

**Characteristics**	
Age, years, median (IQR)	55 (41–65)
Female, *n* (%)	282 (52.6)
Proportion of BM blasts, %, median (IQR)	62 (42, 80)
Hemoglobin, g/L, median (IQR)	75 (65, 91)
WBC × 10E+9/L, median (IQR)	9.75 (2.79, 45.77)
RBC × 10E+9/L, median (IQR)	2.31 (1.95, 2.87)
PLT × 10E+9/L, median (IQR)	35 (20, 70)
**FAB subtypes**	
M0, *n* (%)	10 (1.9)
M1, *n* (%)	24 (4.5)
M2, *n* (%)	276 (51.5)
M4, *n* (%)	79 (14.7)
M5, *n* (%)	124 (23.1)
M6, *n* (%)	4 (0.7)
Unclassified, *n* (%)	19 (3.5)

### Targeted sequencing

Genomic DNA (gDNA) was extracted from the formalin-fixed paraffin-embedded (FFPE) or fresh BM samples, followed by the targeted sequencing of 38 genes (*ASXL1, BCOR, BCORL1, CBL, CEBPA, CSF3R, DNMT3A, ETV6, EV11, MECOM, EZH2, FLT3, FLT3-ITD, FLT3-TKD, GATA2, HOX11, IDH1, IDH2, JAK2, KIT, KMT2A, KMT2A-PTD, KRAS, MPL, MYC, NF1, NPM1, NRAS, NTRK3, RUNX1, SF3B1, SH2B3, SRSF2, TET2, TP53, TPMT, U2AF1*, and *ZRSR2*) on the Novaseq (Illumina, USA) sequencing platform. The original sequencing was aligned with the human reference genome GRCh37. Single nucleotide variations (SNVs) and insertion and deletion (Indels) were screened by Shanghai Rightongene Biotechnology Co., Ltd. (Shanghai, China) based on the filtering conditions: (1) SNVs or Indels with a mutation allele frequency (MAF) of ≥0.001 in databases of 1,000 genomes project, 1,000 genome East Asian, ExAC all, or ExAC East Asian were removed; (2) SNVs or Indels with a variant allele frequency (VAF) of ≥ 1% were retained; (3) dbSNP (v147) sites present in COSMIC database were retained; and (4) SNPs or Indels including stopgain, stoploss, frameshift, non-frameshift, and splicing sites were retained.

### Establishment of a prognostic risk scoring system for Chinese AML

To establish the training and test cohorts, 536 samples were randomly divided into two groups, the training/validation set (70%) and the test set (30%), respectively. The training set was subjected to 10-fold cross-validation to account for variability and provide risk estimates. The mutated genes (*ASXL1, BCOR, BCORL1, CBL, CEBPA, CSF3R, DNMT3A, ETV6, EV11, MECOM, EZH2, FLT3, FLT3-ITD, FLT3-TKD, GATA2, HOX11, IDH1, IDH2, JAK2, KIT, KMT2A, KMT2A-PTD, KRAS, MPL, MYC, NF1, NPM1, NRAS, NTRK3, RUNX1, SF3B1, SH2B3, SRSF2, TET2, TP53, TPMT, U2AF1*, and *ZRSR2*), rearrangements (*AML1*-*ETO, BCR*-*ABL1, CBFB*-*MYH11, DEK*-*NUP214, KMT2A* rearrangement, *MECOM* rearrangement, *NPM1*-*RAR*α, *NUP98* rearrangement, *PLZF*-*RAR*α, *RUNX1*-*RUNXT1*, and *SET*-*NUP214*), and clinicopathologic features (age, sex, proportion of BM blasts, hemoglobin, WBC, and PLT) were included in the models. Conventional Cox regression was used to train the models for assessing survival with the selected variables by Lasso using the “Glmnet” package. The optimal cutoff values for risk score were determined using the X-tile software (Version 3.6.1, Yale University, USA) (8); thereafter, the patients were divided into favorable, intermediate, and adverse groups.

### Statistical analysis

The statistical analysis was performed using GraphPad Prism software v.6 (GraphPad, San Diego, CA, United States). Kaplan–Meier curves with log-rank tests were used to analyze the OS and PFS of AML patients in different groups. A *P*-value of < 0.05 was considered significant.

## Results

### AML classification according to the 4th and 5th WHO classification

The 5th WHO classification made some changes in the diagnostic criteria of AML, including (1) persons with *KMT2A, MECOM*, and *NUP98* rearrangements and *NPM1* mutation were diagnosed with AML regardless of the percentage of blasts; (2) the definition of AML with *CEBPA* mutation was changed to include both biallelic (biCEBPA) and single mutation of *CEBPA* located in the basic leucine zipper (bZIP) region (smbZIP-*CEBPA*); (3) the previous classification of AML with mutated *RUNX1* was abolished; (4) the classification of AML with myelodysplasia-related changes (AML-MRC) has been changed to AML-MR, and the mutations in *ASXL1, BCOR, EZH2, SF3B1, SRSF2, STAG2, U2AF1*, and *ZRSR2* genes were newly defined as the defining cytogenetic abnormalities; (5) the classification of AML with other defined genetic alterations was newly added; and (6) the classification of AML, not otherwise specified (NOS) was replaced with AML, defined by differentiation.

**Figure 1:**
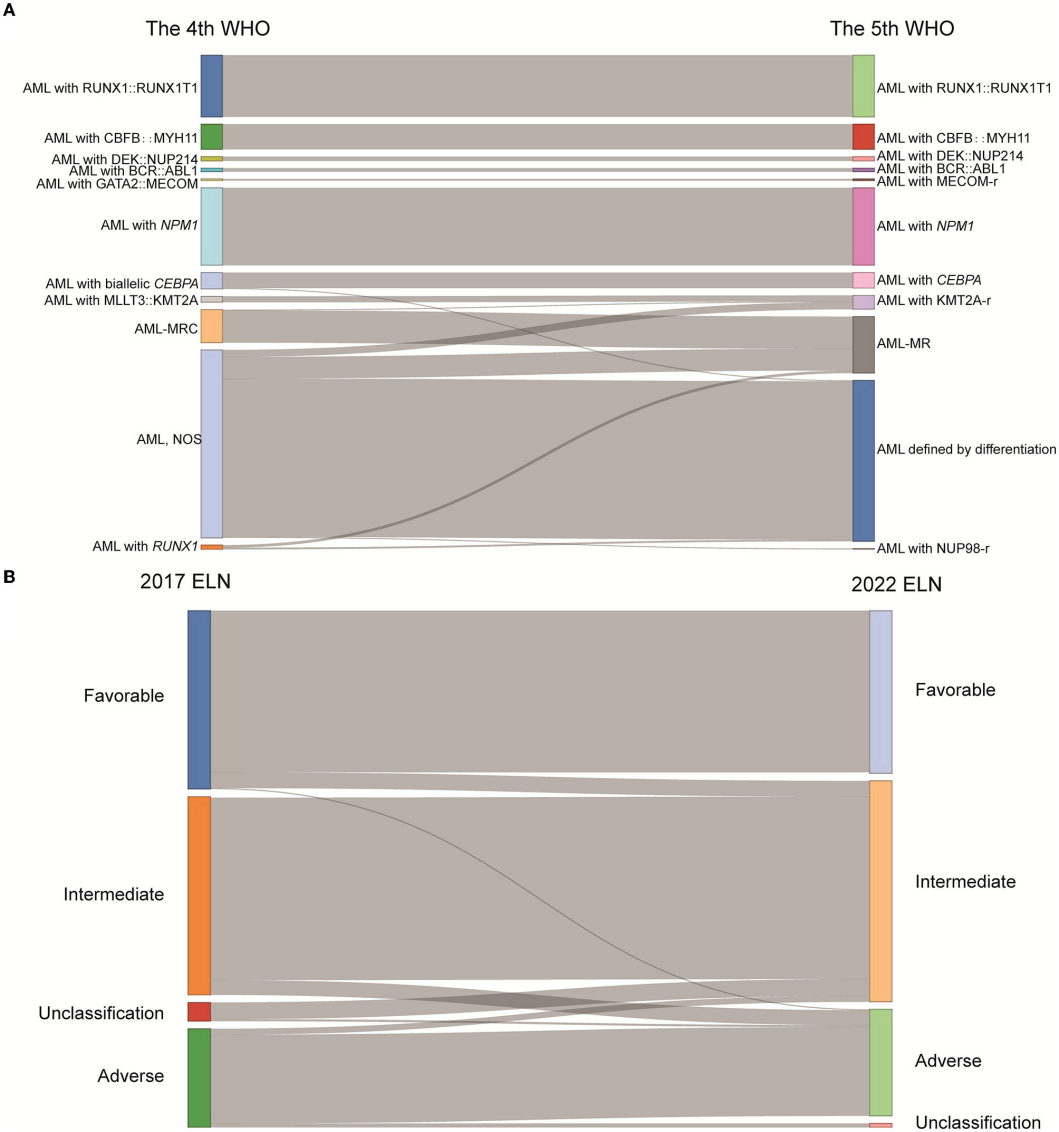
The classification of AML patients based on the WHO and ELN guidance. **(A)** Sankey diagram demonstrated the relationship between AML patients' subtypes defined in the 4th and 5th WHO classifications. **(B)** Sankey diagram demonstrated the relationship between AML patients' subtypes defined in the 2017 ELN and 2022 ELN guidance (WHO, World Health Organization; ELN, European Leukemia Network; AML, acute myeloid leukemia; *MECOM*-r, *MECOM* rearrangement; *KMT2A*-r, *KMT2A* rearrangement; *NUP98*-r, *NUP98* rearrangement; AML-MRC, AML with myelodysplasia-related changes; AML-MR, AML, myelodysplasia-related; NOS, not otherwise specified).

A total of 485 and 487 of the 536 patients were classified according to the 4th and 5th WHO classifications, respectively, as the mutation location of *CEBPA* or the VAF of *FLT3-ITD* is not available. Six subgroups, AML with RUNX1::RUNX1T1 (*n* = 70), AML with CBFB::MYH11 (*n* = 29), AML with DEK::NUP214 (*n* = 5), AML with BCR::ABL1 (*n* = 4), AML with GATA2::MECOM (*n* = 2), and AML with *NPM1* (*n* = 89), remained unchanged according to the 5th WHO, although the subgroup of AML with GATA2::MECOM was changed to AML with *MECOM* rearrangement. The group that changed the most was the AML, NOS (not otherwise specified) subgroup (*n* = 212); in detail, 8 patients were divided into the AML with *KMT2A*-rearrangement subgroup, 25 patients were divided into the AML-MR subgroup, and one case was divided into the AML with *NUP98*-rearrangement subgroup based on the 5th WHO classification (Figure 1A, Table 2).

**Table 2:** Re-stratification matrix of the number of AML patients classified in each of the 4th WHO 2016 classification and each of the 5th WHO classification (*n* = 485).

**4th WHO**	**AML withRUNX1::RUNX1T1**	**AML withCBFB::MYH11**	**AML withDEK::NUP214**	**AML withBCR::ABL1**	**AML withGATA2::MECOM**	**AML withMLLT3::KMT2A**	**AML with*NPM1***	**AML with biallelic *CEBPA***	**AML with *RUNX1***	**AML-MRC**	**AML-NOS**
**5th WHO**											
AML with RUNX1::RUNX1T1	71	0	0	0	0	0	0	0	0	0	0
AML with CBFB::MYH11	0	29	0	0	0	0	0	0	0	0	0
AML with DEK::NUP214	0	0	5	0	0	0	0	0	0	0	0
AML with BCR::ABL1	0	0	0	4	0	0	0	0	0	0	0
AML with MECOM-r	0	0	0	0	2	0	0	0	0	0	0
AML with KMT2A-r	0	0	0	0	0	7	0	0	0	1	8
AML with NUP98-r	0	0	0	0	0	0	0	0	0	0	1
AML with *NPM1*	0	0	0	0	0	0	89	0	0	0	0
AML with biallelic *CEBPA*	0	0	0	0	0	0	0	18	0	0	0
AML-MR	0	0	0	0	0	0	0	0	3	37	25
AML defined by differentiation	0	0	0	0	0	0	0	1	2	0	182

AML, acute myeloid leukemia; MECOM-r, MECOM rearrangement; KMT2A-r, KMT2A rearrangement; NUP98-r, NUP98 rearrangement; AML-MRC, AML with myelodysplasia-related changes; AML-MR, AML, myelodysplasia-related.

### Risk stratification of AML according to the 2017 and 2022 ELN guidelines

Diagnostic criteria were largely unchanged in the new proposal, except for the following: (1) persons with *FLT3-ITD* were classified as intermediate regardless of the VAF; (2) the previously defined favorable risk of biCEBPA was changed to bZIP in-frame mutated *CEBPA*; (3) KAT6A::CREBBP and mutated *ASXL1, BCOR, EZH2, RUNX1, SF3B1, SRSF2, STAG2, U2AF1*, or *ZRSR2* were added as adverse events; and (4) VAF ≥ 10% was defined as an additional condition for the adverse event of *TP53* mutation.

A total of 483 and 498 patients were submitted to risk stratification based on the 2017 and 2022 ELN guidance, respectively. The stratification for most patients (442/501) according to the 2022 ELN guidance was consistent with the 2017 ELN. In contrast, 16 patients in the favorable group based on the 2017 ELN guidance were regrouped into the intermediate group based on the 2022 ELN guidance due to the low VAF value of *FLT3-ITD* and the lack of the bZIP in-frame mutated *CEBPA*. In addition, 13 patients in the intermediate group based on the 2017 ELN guidance were regrouped into the adverse group based on the 2022 ELN guidance due to the mutations of *BCOR, SRSF2, U2AF1*, and *ZRSR2*. Seventeen unclassified patients (the detailed information of *CEBPA* and *FLT3-ITD* unknown) based on the 2017 ELN guidance were regrouped into the intermediate group (Figure 1B, Table 3).

**Table 3:** Re-stratification matrix of the number of AML patients classified in each of the 2017 ELN classification and each of the 2022 ELN classification (*n* = 501).

**2017 ELN**	**Favorable**	**Intermediate**	**Adverse**	**Unclassification**
**2022 ELN**				
Favorable	164	0	0	0
Intermediate	16	186	6	17
Adverse	1	13	92	2
Unclassification	0	0	4	0

### Prognosis analysis according to the WHO classification and ELN guidance

**Figure 2:**
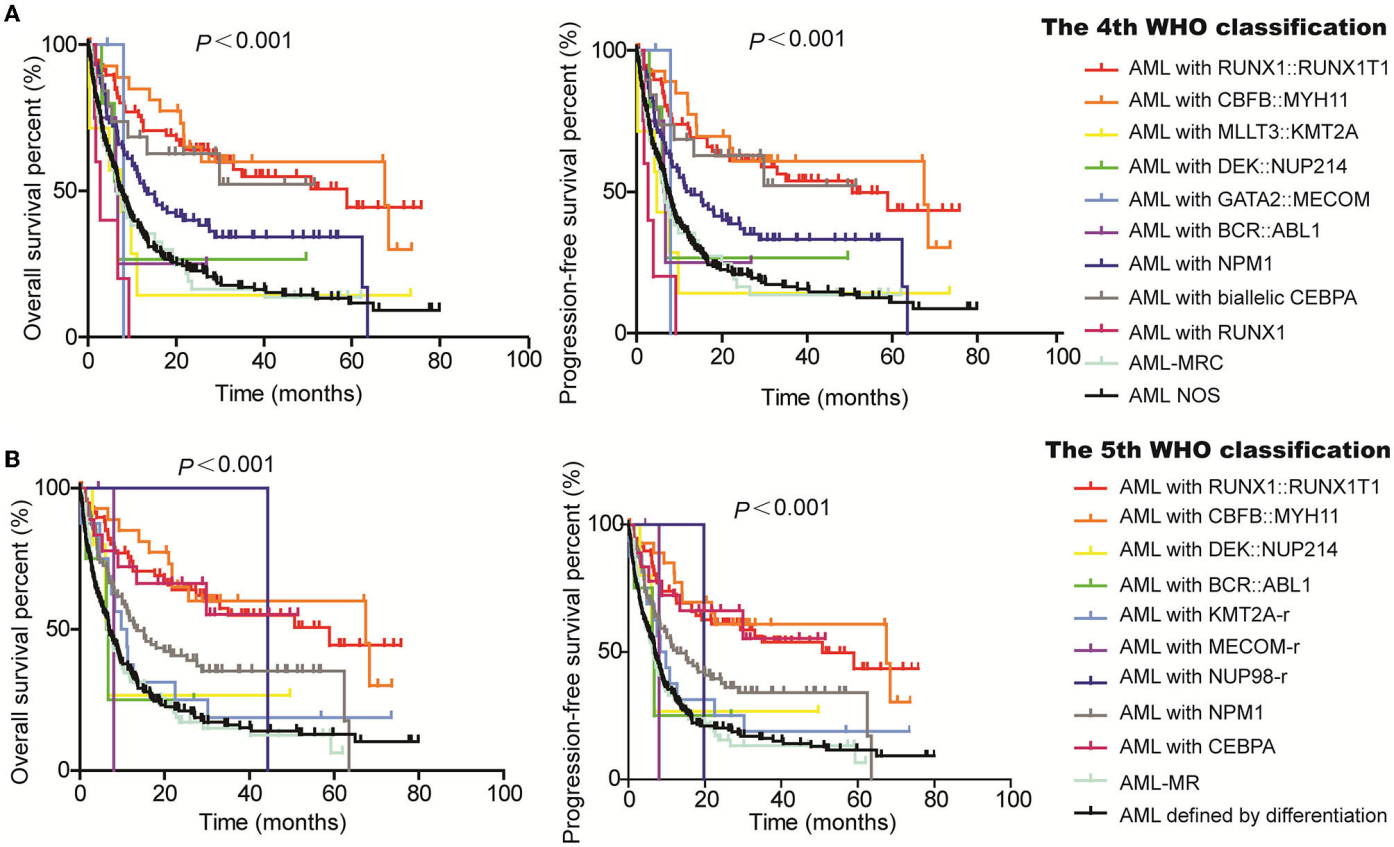
Assessment of the value of 4th and 5th WHO classifications on the PFS and OS of AML patients. Kaplan–Meier curves were used to assess the OS and PFS of patients of the favorable, intermediate, and adverse groups according to the 4th **(A)** and 5th WHO classifications [**(B)**; WHO, World Health Organization; AML, acute myeloid leukemia; *MECOM*-r, *MECOM* rearrangement; *KMT2A*-r, *KMT2A* rearrangement; *NUP98*-r, *NUP98* rearrangement; AML-MRC, AML with myelodysplasia-related changes; AML-MR, AML, myelodysplasia-related; NOS, not otherwise specified].

**Figure 3:**
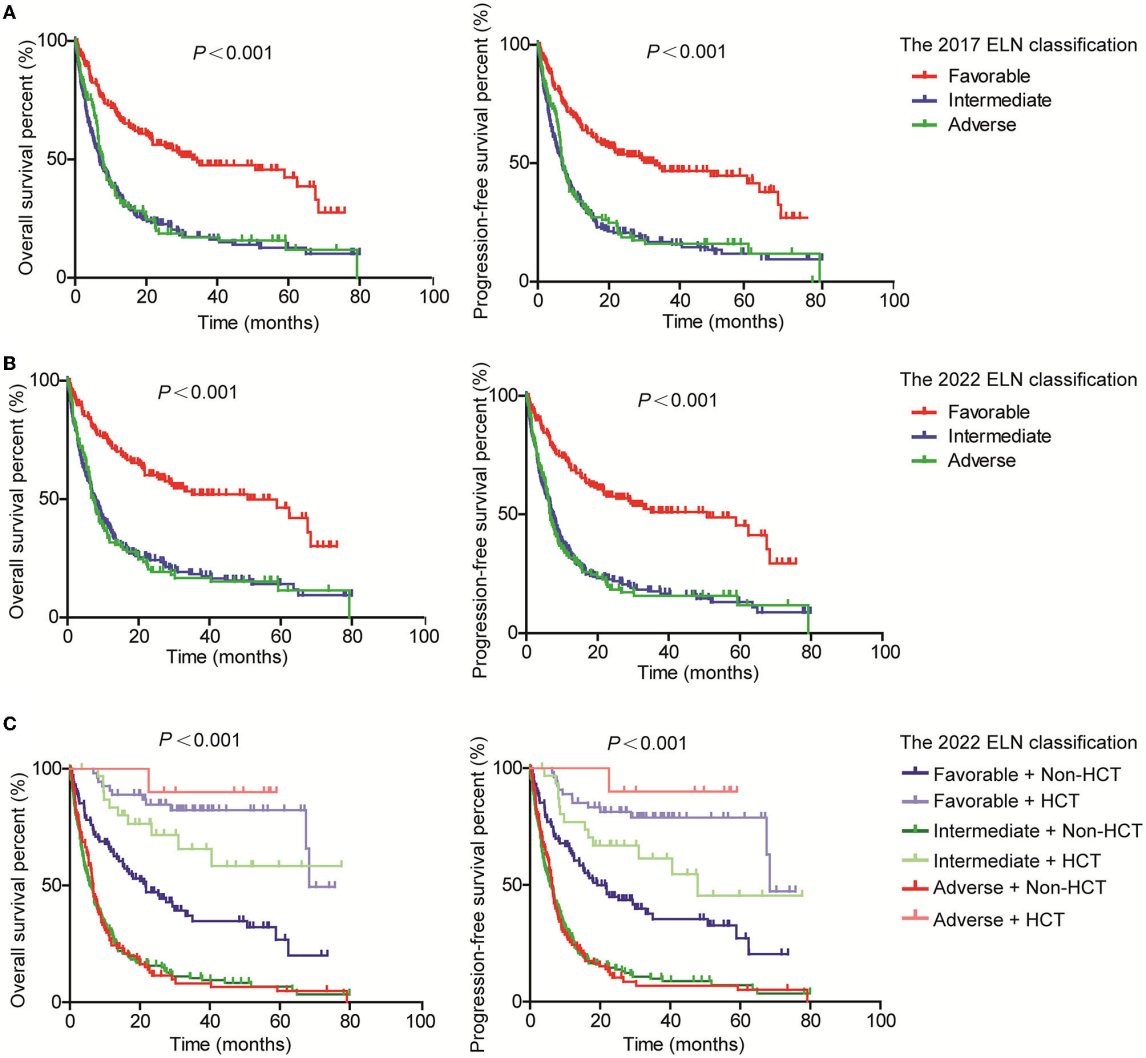
Assessment of the value of 2017 and 2022 ELN guidance on the PFS and OS of AML patients. Kaplan–Meier curves were used to assess the OS and PFS of patients of the favorable, intermediate, and adverse groups according to the 2017 **(A)** and 2022 **(B)** ELN guidance, together with transplantation [**(C)**; ELN, European Leukemia Network; HCT, hematopoietic stem cell transplantation].

Moreover, we evaluated the PFS and OS of subgroups based on the WHO classification and ELN guidance. Both PFS and OS rates were higher in AML patients with RUNX1::RUNX1T1, CBFB::MYH11, and bi*CEBPA*, while the PFS and OS rates were lower for patients with MLLT3::KMT2A and GATA2::MECOM according to the 4th WHO classification (Figure 2A). Similarly, the PFS and OS rates for patients with RUNX1::RUNX1T1, CBFB::MYH11, and bi*CEBPA* were higher, and the prognosis for patients of the AML-MR and AML defined by differentiation subgroups was worse according to the 5th WHO classification (Figure 2B). From the ELN subgroups, both PFS and OS curves of patients of the intermediate and adverse groups were not very distinguishable according to the 2017 (Figure 3A) and 2022 ELN guidance (Figure 3B), although the OS and PFS curves for the favorable group and other groups could be distinguished well. Also, we assessed whether the therapeutic means affected the prognosis of AML patients. Hematopoietic stem cell transplantation (HCT) significantly improved the PFS and OS of patients in favorable, intermediate, and adverse groups as compared with the non-HCT patients. However, the PFS and OS curves for the non-HCT patients of the intermediate and adverse groups remained not very distinguishable (Figure 3C). These results affirmed the clinical value of both WHO and ELN, but a better prognosis model should be established in Chinese cohorts.

### Establishment of the prognosis models for Chinese AML patients

**Figure 4:**
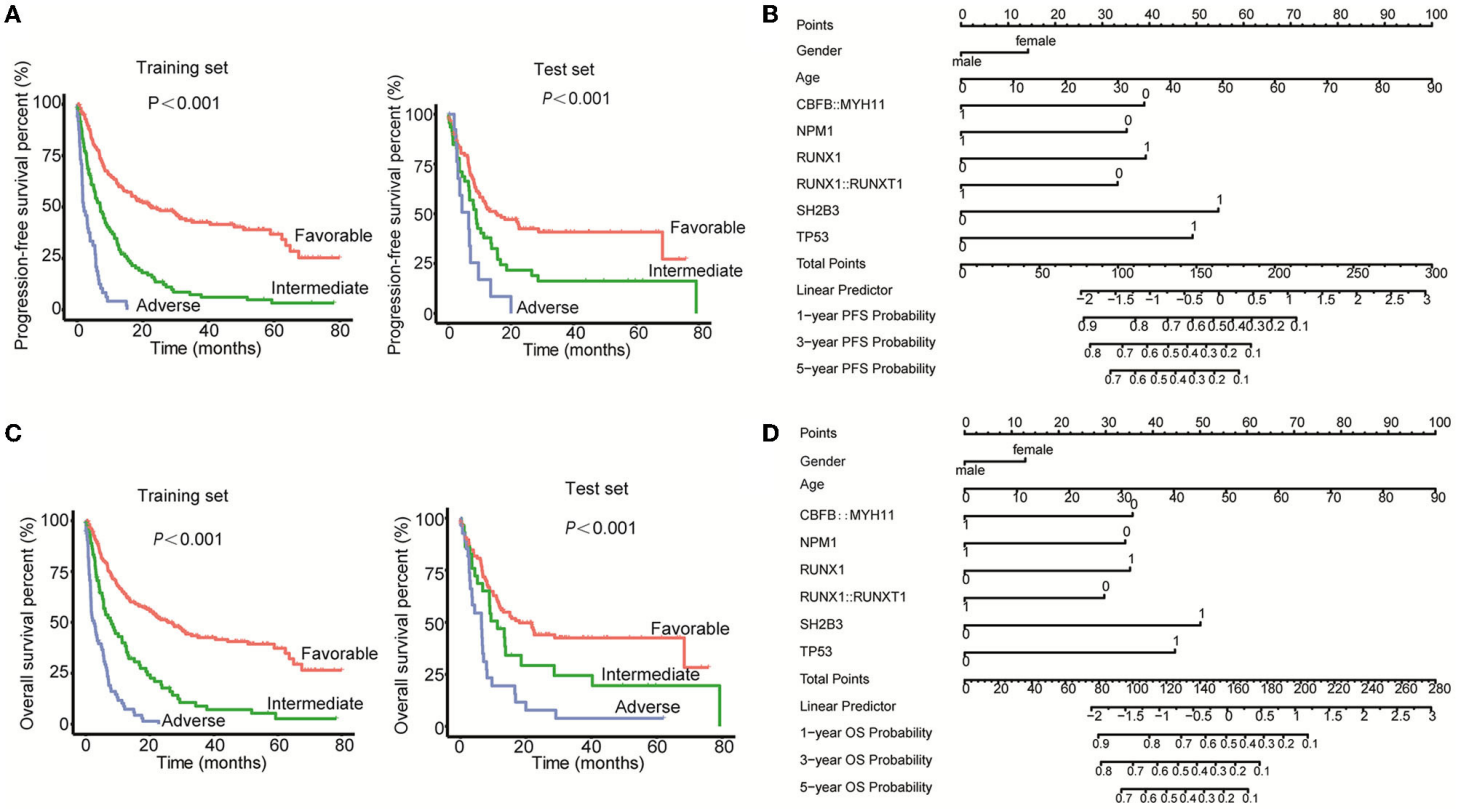
Establishment of the prognosis models for Chinese AML patients. **(A)** Kaplan–Meier curves were used to assess the PFS of patients of the favorable, intermediate, and adverse groups in both the training set and test set. **(B)** The nomogram model was applied to demonstrate the contributions of the selected factors to the 1-, 3-, and 5-year PFS probability. **(C)** Kaplan–Meier curves were used to assess the OS of patients of the favorable, intermediate, and adverse groups in both the training set and the test set. **(D)** The nomogram model was applied to demonstrate the contributions of the selected factors to the 1-, 3-, and 5-year OS probability.

To establish a better prognosis model for Chinese patients with newly diagnosed AML, mutation, rearrangements, and clinicopathologic features were considered. For the PFS model, risk score was first calculated according to the following formula, risk score = 0.324134^*^sex (male = 1, female = 2) + 0.025315^*^age- 0.88687^*^CBFB:: MYH11-0.801312^*^NPM1+0.89387^*^RUNX1-0.757403^*^RUNX1::RUNX1T1+1.244844^*^SH2B3+1.120662^*^TP53, in which positive was defined as “1” and negative was defined as “0” in terms of gene mutation and fusion. Then, the patients were divided into three groups, favorable (risk score ≤ 1.80), intermediate (risk score < 1.8 < 2.37), and adverse (risk score ≥ 2.37). As shown in Figure 4A, the PFS time of the favorable group was obviously longer than the intermediate and adverse groups, as well as the intermediate group vs. the adverse group in both training and test sets. Furthermore, the nomogram demonstrated the contributions of the selected factors to the 1-, 3-, and 5-year PFS probability (Figure 4B).

For the OS model, the risk score was first calculated using the formula = 0.319677^*^sex (male = 1, female = 2) + 0.027509^*^age- 0.883355^*^CBFB::MYH11-0.84456^*^NPM1+0.870257^*^RUNX1-0.73479^*^RUNX1::RUNX1T1+1.240181^*^SH2B3+1.106195^*^TP53. After calculating the risk score of each patient, the patients were divided into three groups, favorable (≤ 1.94), intermediate (< 1.94 < 2.33), and adverse (≥2.33). The PFS time of the favorable group was obviously higher as compared with the intermediate and adverse groups, as well as the intermediate group vs. the adverse group in both the training set and test set (Figure 4C). In addition, the nomogram demonstrated the contributions of the selected factors to the 1, 3, and 5-year OS probability (Figure 4D). Collectively, we constructed a prognosis model for Chinese AML patients through the integration of the mutations, fusions, and clinical information.

## Discussion

Recently, the 5th WHO guidance was issued, which has made some modifications to the classification of hematologic malignancies including AML. Herein, we compared the 4th and 5th WHO in the Chinese AML classification. The biggest change was that 25 (5.2%), 8 (1.6%), and 1 (0.2%) patients in the AML, NOS group according to the 4th WHO, were re-classified into the AML-MR, KMT2A rearrangement, and NUP98 rearrangement subgroups, respectively. Still, 38% (185/486) of patients were divided into the AML defined by differentiation subgroup according to the 5th WHO classification due to the limitation of sequencing technology earlier in the years. However, it was decreased compared to the proportion of AML, NOS (44.5%, 216/485). With the advances in gene detection technologies, patients are divided into more precise categories with clearer treatment strategies and prognoses (9–11). For instance, the mutations of *ASXL1, BCOR, EZH2, SF3B1, SRSF2, STAG2, U2AF1*, and *ZRSR2* genes are added as the defining cytogenetic abnormalities, and patients carrying one of which are considered a member of the AML-MR subgroup, while the subgroup defined by *RUNX1* mutation is abolished. It has been reported that some somatic mutations, including *ASXL1, BCOR, EZH2, SF3B1, SRSF2, STAG2, U2AF1*, and *ZRSR2* mutations are associated with an adverse prognosis of AML (12–14). *RUNX1* mutations were previously reported to be linked to unfavorable outcomes in AML patients (15). However, increasing evidence has demonstrated that normal *RUNX1* is also implicated in leukemogenesis. Sood et al. (16) indicated that leukemic cells of core-binding factor AML and certain types of leukemia with *KMT2A* rearrangements require normal *RUNX1* to survive. Wesely et al. (17) showed that *RUNX1* was of importance in maintaining leukemia stem cells across various genetic subgroups in AML. Thus, either mutations or normal *RUNX1* is essential in AML development.

In addition, we compared the risk stratification of AML patients according to the 2017 and 2022 ELN guidance. In total, 16 (3.3%) patients in the favorable group and 13 (2.7%) patients in the intermediate group were re-classified to the intermediate and adverse groups based on the 2022 ELN guidance, while 6 (1.2%) patients in the adverse group were re-grouped into the intermediate group based on the 2022 ELN guidance. Regrettably, both the PFS and OS curves of the intermediate and adverse groups were not very distinguishable in our cohort even after excluding the patients who received HCT, which significantly improved the prognosis of AML, as previously reported (18, 19). This result further highlights the high heterogeneity of AML, but it also cannot exclude the reasons for inadequate detection means as early as 2013 and the small sample size.

Based on the above results, we constructed the prognosis model for Chinese AML patients through the integration of mutations, rearrangements, and clinicopathologic features. Finally, the mutations of *NPM1, RUNX1, SH2B3*, and *TP53* genes, fusions of CBFB::MYH11 and RUNX1::RUNX1T1, and the clinical factors of sex and age were selected as the important influencing factors of both PFS and OS. Among them, CBFB::MYH11, RUNX1::RUNX1T1, and *NPM1* mutations were the protective factors, while the mutations of *RUNX1, SH2B3*, and *TP53* genes were adverse factors, which were consistent with the ELN guidance (6, 7). In addition, we found that older adults and female patients were also two adverse factors of PFS and OS for Chinese AML patients. Stabellini et al. (20) recently studied the effect of sex on the survival of adults with AML and found that male patients had a lower risk of death than female patients (aHR = 0.41) in a total of 1,020 AML patients (57.4% male patients). This was consistent with our study, which demonstrated that being male was a protective factor for both PFS and OS in Chinese AML patients. In addition, Bin et al. (21) showed that age served as an independent prognostic factor to predict the 1-, 3-, and 5-year survival of AML patients, which was consistent with our study.

Collectively, this study compared the 4th and 5th WHO, as well as the 2017 and 2022 ELN guidance in Chinese AML patients. Although the classification and risk stratification were improved and defined by the 5th WHO classification and 2022 ELN guidance, the risk models were not very suitable. Based on this, we established a risk model for Chinese AML patients, which included age, sex, mutations (*NPM1, RUNX1, SH2B3*, and *TP53*), and fusions (CBFB::MYH11 and RUNX1::RUNX1T1). This model could easily help divide the patients into favorable, intermediate, and adverse groups, which may be suitable for Chinese AML patients.

## Data availability statement

The data presented in the study are deposited in the Genome Sequence Archive for Human (GSA-Human) repository, accession number “HRA004529”, https://ngdc.cncb.ac.cn/gsa-human/browse/HRA004529.

## Ethics statement

The studies involving human participants were reviewed and approved by Ethics Committee of the First Affiliated Hospital of Xi'an Jiaotong University. Written informed consent to participate in this study was provided by the participants' legal guardian/next of kin.

## Author contributions

XW and JieW wrote the paper. SW, JuZ, JiZ, DW, ML, SZ, YC, HL, HZ, JinW, and WW provided the data. BX and GL analyzed the data. HW, HX, and PH reviewed the paper. All authors contributed to the article and approved the submitted version.
